# Parent attitudes, family dynamics and adolescent drinking: qualitative study of the Australian parenting guidelines for adolescent alcohol use

**DOI:** 10.1186/1471-2458-12-491

**Published:** 2012-06-29

**Authors:** Conor Gilligan, Kypros Kypri

**Affiliations:** 1 Discipline of Health Behaviour Science and Priority Research Centre for Health Behaviour, School of Medicine and Public Health, University of Newcastle, University Drive Callaghan, Newcastle, 2308, NSW, Australia; 2 Centre for Clinical Epidemiology & Biostatistics, School of Medicine and Public Health, University of Newcastle, University Drive Callaghan, Newcastle, 2308, NSW, Australia

**Keywords:** Parent, Adolescent, Alcohol, Supply, Guidelines

## Abstract

**Background:**

Parents play a critical role in their children’s introduction to alcohol. A range of parenting factors have been associated with the progression to risky drinking among adolescents, and have recently formed the basis of the Australian ‘Parenting Guidelines for Adolescent Alcohol Use’ designed to help parents delay or reduce their adolescents’ alcohol use.

**Methods:**

This study aimed to explore the experiences and attitudes of parents of adolescents to gain insight into: (1) the extent to which the behaviours of parents follow the recommendations made in the guidelines; and (2) approaches to reduce hazardous drinking among adolescents. Thirty-two telephone and face-to-face interviews were conducted with parents, and the content of discussions was examined using thematic analysis.

**Results:**

Parents used approaches they thought would minimise harm and promote healthy development in their children. The guidelines address key areas of concern for parents but their adherence to these approaches is low in certain areas. Many parents provided some alcohol to their adolescents and often cited the social norm of drinking among their adolescents’ peers as a source of pressure to supply.

**Conclusions:**

Further dissemination of the guidelines may be the first step in a public health strategy, but it is likely that parents will require support to effectively adopt the recommendations. Understanding the influences on parents’ beliefs about their children’s drinking and the functions of social networks in the creation of behavioural norms relating to alcohol consumption and supply may be necessary to address adolescent risky drinking.

## Background

Children are exposed to and learn about alcohol from an early age from their parents and families, the wider community and the media. Children recognise alcoholic beverages and develop an attitude towards alcohol from as early as pre-school [1]. While there is a shift in emotional attachment during early adolescence, there is evidence to support the continuing influence of parents on development [2] through late adolescence and into early adulthood [3]. Parents often give their children alcohol with the intention to encourage a safe introduction to drinking, and to control what and how much they drink [4]. A recent review performed by the authors calls for research into, among other things, the volume and context of parental supply of alcohol [5].

In a systematic review of longitudinal studies investigating parenting factors associated with adolescent drinking, Ryan et al. identified several predictors of delayed alcohol initiation and lower levels of later drinking [6]. Key parental factors included parental modeling of drinking behaviour, alcohol-specific communication, disapproval of adolescent drinking, general discipline, rules about alcohol, parental monitoring, parent-child relationship quality, family conflict, parental support, parental involvement, and general communication [6].

These risk and protective factors were explored in a Delphi consensus study [7] and have since formed the basis of the ‘Parenting Guidelines for Adolescent Alcohol Use’ (hereafter referred to as the guidelines) designed to help parents delay or reduce their adolescent’s alcohol use as recommended by the Australian National Health and Medical Research Council (NHMRC) (http://www.parentingstrategies.net[8]). While legislation concerning secondary supply of alcohol exists in most Australian states, and the NHMRC recommends delaying initiation of drinking for as long as possible for 15 to 17 year-olds [9], this information is not widely disseminated to parents. It appears that many parents fail to follow this advice and often provide adolescents with alcohol as a harm minimisation measure with the aim of controlling the type and quantity of alcohol they consume [10,11]. How well the normal practices of Australian parents fit with the more extensive guideline recommendations is unclear. In all Australian jurisdictions, purchase of alcohol under 18 years of age is illegal. The laws regarding who can supply alcohol to minors vary by jurisdiction. In NSW parents/guardians or other adults who have the permission of parents/guardians can legally supply alcohol to children under 18 years in private settings (NSW Liquor Act, 2007).

The aim of this study was to explore the experiences and attitudes of parents of adolescents in a community sample to gain insight into: (1) the extent to which the behaviours of parents follow the recommendations made in the guidelines; and (2) parents’ approaches to adolescent drinking and alcohol initiation.

## Methods

We adopted a qualitative approach, conducting on-one-one interviews with parents to capture as much as possible, parents’ honest views and opinions. Individual interviews were seen as more appropriate than focus group discussions, as they reduced the potential for contamination through group processes, or reluctance to share due to social stigma. The study was designed to capture rich information about parents’ experiences and attitudes to enable a thorough analysis of the decisions made by parents, and their justification for these decisions, and to evaluate this information in relation to its adherence or otherwise with the guidelines.

Parents were recruited through posters displayed in local cafés and at the University of Newcastle, and through University of Newcastle staff networks. Parents were invited to contact the researcher to arrange a time and place for a face-to-face or telephone interview. Those responding to the invitation were also encouraged to disseminate it to their own networks. To be eligible, respondents were required to be the parent or guardian of a child aged 13-17 years. Eligibility criteria were explained in invitation posters, and assessed in initial contact with respondents.

A semi-structured interview schedule was developed, based on the issues emerging from current literature and the guidelines. Parents were asked about their own alcohol consumption, the nature of their relationships with their children, the behavior and attitudes of their children in relation to alcohol, their behaviours in terms of supply of alcohol, attitudes toward their children drinking, and household rules about alcohol consumption. These question areas were largely based on assumptions derived from the systematic review of longitudinal studies by Ryan et al. (2010). The interview schedule is attached as Additional file 1.

### Qualitative data analysis

Each interview, conducted by CG or a research assistant was conducted according to the semi-structured interview schedule. Recruitment was continued until data saturation was reached. Interviews were audio recorded to ensure the accuracy of notes taken, but were not transcribed verbatim. Extensive notes were taken during and after each interview, enabling data reduction and preliminary analysis of parental adherence to the guidelines. Discussions were held between the authors after initial review of individual interview notes, and again after each author had evaluated parents’ adherence to the guidelines. The authors were generally in agreement at both steps, though some additional points and interpretation of parents’ statements were added at the first discussion step. Authors agreed about the categories into which parental adherence to the guidelines fell, and thus proceeded to group the behaviours and guidelines into three categories as described below.

### Ethical approval

Ethical approval was granted by the University of Newcastle Human Research Ethics Committee (Approval number H-2010-1144). Each participant was provided with an information statement and was asked to sign a consent form prior to participating. All participants received a $30 shopping voucher toward the costs associated with their participation.

## Results and discussion

### Participants

Twenty face-to-face, and twelve telephone interviews were conducted at times and places convenient for each participant, and each lasted approximately one hour. Parents had an average of 2.4 children, and the child/ren on whom they were reporting were aged 15 on average. The demographic characteristics of participants are summarized in Table 1.

**Table 1:** Characteristics of participating parents

**Characteristic**	**N (%) or mean (SD)**
**Gender of the parent**	
- Male	5 (16%)
- Female	27 (84%)
Age	
- 35-44	10 (31%)
- 45-54	19 (59%)
- 55-64	2 (6%)
- 65 or over	1 (3%)
**Marital status**	
- Married	25 (78%)
- Divorced/Separated/Single	7 (22%)
**Parents education**	
- Less than yr 10	1 (3%)
- Completed yr 10	5 (16%)
- Completed Yr 12	8 (25%)
- Tertiary diploma or degree	9 (28%)
- Post graduate degree/s	8 (25%)
**Employment**	
- Full time/part time	28 (88%)
- Unemployed	2 (6%)
- Retired	1 (3%)
- Full-time study	1 (3%)
**Australian born**	22 (69%)
Overseas born (one Canada, one Netherlands, one Fiji, one Ireland, two New Zealand, one South Africa, two USA and one Zimbabwe)	10 (31%)
Language spoken at home	
- English only	31 (97%)
- English and another language	1 (3%) - Hindi

Parents’ frequency of alcohol consumption ranged from ‘never’ through to ‘four or more times a week’. Average consumption was between 2-4 times a month and 2-3 times a week, with parents typically drinking 1-4 standard drinks per occasion. Parents generally reported having more than four drinks on one occasion never or less than monthly.

### Adherence to guidelines

The Parenting for Adolescent Alcohol Use are summarized in Table 2. We did not ask parents about their knowledge of or adherence to the guidelines. Most topics covered in the guidelines were, however, raised by participants in almost all interviews, and many parents reported behaviours that were consistent with the guidelines. Parents’ behaviours in relation to the guidelines fell into three categories:

(a) Those followed reasonably closely, with some deliberate thought (parent-child relationships, parental monitoring);

(b) Those often followed but not necessarily in a conscious fashion (household rules, communication about alcohol, parental modeling); and

(c) Those followed less often and apparently more difficult for parents to adhere to (protection from peer influence and supply of alcohol).

**Table 2:** Summary of the parenting guidelines for adolescent alcohol use (www.parentingstrategies.net)

**Delay your adolescent’s introduction to drinking alcohol**	Aim to keep your adolescent child from experimenting with alcohol for as long as possible. Do not give them any alcohol while they are under the age of 15, and delay their first alcoholic drink for as long as possible
**Model responsible drinking and attitudes towards alcohol**	Model responsible drinking by establishing and following your own rules for drinking responsibly. Tips include: - Limit your alcohol use, especially in front of your children
	- Do not get drunk, especially in front of your children
	- Sometimes decline the offer of alcohol - Provide food and non-alcoholic beverages if making alcohol available to guests
	- Never drink and drive
	- Do not let other adults drive after they have been drinking
	- Do not convey to your children the idea that alcohol is fun or glamorous through
	- stories about your own or others’ drinking
	- Do not portray alcohol as a good way to deal with stress, such as by saying, “I’ve had a bad day, I need a drink!”
	- Use healthy ways to cope with stress without alcohol, such as exercise, listening to music, or talking things over
**Talk to your child about alcohol**	- Before talking to your child, take some time to prepare for the conversation.
	- What to talk about: Don’t present a permissive approach, talk about alcohol-related harms, the health benefits of choosing not to drink, and explain that their brain is still developing and is therefore more vulnerable to harm caused by alcohol.
	- Emphasise the short-term harms associated with alcohol.
	- Discuss perceptions: Ask your child what they think about alcohol. Ask them why they think young people drink.
	- Your expectations (with older adolescents): Discuss how, if your adolescent does drink, they should do so in moderation.
	- Explain your expectations for specific situations, such as at family celebrations, adolescent parties or "Schoolies Week". Discuss how risks associated with alcohol can be minimised
**Establish family rules**	- Involve your adolescent in developing family rules for them to follow. Once established, make sure the family is clear on exactly what the rules are and that each member understands them.
	- Be prepared to negotiate on rules regarding minor matters, but do not change the family rules or consequences without first discussing it with your adolescent.
	- Review rules as your adolescent shows more maturity and responsibility.
	- Parents should support each other regarding family rules and present a united front in enforcing them.
	- Establish realistic consequences for when family rules are broken. Enforce established consequences consistently every time that family rules are broken
**Monitor your adolescent when you are not around**	- Before your adolescent goes out, you should: - Ask them where they will be, what they will be doing, and who they will be with - Set a curfew and know what time to expect them home - Make arrangements with them about how they will get home safely - Ask them to contact you if their plans change - Make sure they have a way to contact you If giving them money, discuss how much they will need and how it will be spent
**Prepare your adolescent to deal with the influence of peers**	Encourage positive friendships, enlist the support of other parents, and help them deal with peer pressure to drink
**Preparing for unsupervised adolescent drinking**	- Discuss situations adolescents may be faced with where other people are misusing alcohol.
	- Help your adolescent to develop strategies for handling or removing themselves from situations involving alcohol misuse – offer to pick them up, talk about ways to minimise any potential embarrassment that may be associated with getting picked up.
	
	- Discuss drink spiking and other dangers
**Establish and maintain a good relationship with your adolescent child**	There are a number of things you can do to establish and maintain a good relationship with your adolescent, such as:
	-Support them in pursuing their interests and in dealing with problems
	- Show an interest and be involved in their life
	- Work to create open communication between yourself and your adolescent
	- Cultivate their trust by being consistent in following through on promises and enforcing rules
	- Regularly demonstrate that you care about them
	- Regularly tell them that you love them

Here we present the results of our interviews with parents according to these three categories, and explore the results, their adherence to the guidelines, and what existing literature suggests about each issue.

### Closely followed rules

#### Establish and maintain a good relationship with your adolescent child

Central for most of the parents we interviewed was a desire to maintain a good relationship with their child, and encourage communication. While each of the parents had their own approach to maintaining a good relationship with their child, in general, the approaches taken closely matched the suggestions made in the guidelines. For example, parents attempted to talk to their children, showed interest in their lives, and encouraged them to be open about the presence of alcohol at parties and in other social situations, in order to cultivate trust. These sentiments are reflected in the following comments:

"“its important to keep the communication lines open” and “the bottom line is that you have to trust them” (Mother of a 16 year-old girl);"

"“it happens anyway so I would rather they tell me” (Mother of a 14 year-old girl and 16 and 18 year-old boys); and"

"“We have a good relationship with their friends…we try to make it pretty cool for them to hang out at our place” (Mother of 13 year-old girl and 15 year-old boy)."

Several studies show that family relations and the strength of the parent-child bond can affect drug use, both directly and indirectly through an influence on the choice of friends [12-14]. There is conflicting evidence on the impact of parental attachment on adolescent drinking. Van der Vorst et al. found that good attachment was not protective against adolescent alcohol consumption, but that strict parental control did have a protective effect [15]. Ryan et al. cited evidence that parental involvement is associated with delayed alcohol initiation but not with lower levels of later use, and the reverse for parental support i.e. there was no association with age of initiation but parental involvement was predictive of lower levels of later use [6]. Parent-child relationship quality is reportedly associated with both delayed alcohol initiation and reduced levels of later use [6].

#### Monitor your adolescent when you are not around

Parental behaviour in relation to parental monitoring matched reasonably well with the guidelines. Parents generally attempted to know where their children were, and who they were with. Often, parents used dropping-off and picking-up children as a way of monitoring their whereabouts and behaviours. Many parents acknowledged that it is difficult to monitor what happens at parties and other events, but they focused on building a trusting relationship whereby their adolescent communicates with them about such events. One mothers’ comment *“I want them to feel that they can talk to me”* (Mother of a 17 year-old boy and 15 year-old girl) echoed the sentiments of many. A harm minimization approach was mentioned by several parents, ensuring their children could get home safely, discouraging drink driving, and making themselves *“accessible 24-7”* if needed. Messages such as *“never get in a car with someone who has been drinking”* (Mother of 13, 16 and 18 year-old boys) were common. One mother (of a 13 year-old girl and 16 year-old boy) talked about telling her children *“No mucking around, if you are out of your depth, I can be there in a flash to pick you up”.*

Previous research suggests that parental monitoring and supervision can prevent or delay the onset of adolescent substance use (including alcohol) [16]. In a longitudinal study of drug taking among year 8-10 students (aged 13-16) it was found that higher levels of monitoring were protective against the misuse of drugs, even when exposed to drug-using peers [17]. In relation to alcohol, Ryan et al. reported that greater parental monitoring is associated with earlier initiation, but lower levels of later alcohol use [6]. This result suggests that parental monitoring may occur in a context of early initiation to alcohol in the home, which is consistent with findings that early initiation of alcohol is likely to occur when children are allowed to drink at home [18,19] or at family gatherings [20].

### Guidelines followed to some extent

#### Establish family rules

While some families did have rules that matched with those suggested in the guidelines, these rules were rarely planned or established through family discussion as the guidelines recommend. In further discordance with the guidelines, parents often expressed a reluctance or incapacity to punish their children, and consequences were rarely enforced if household rules were broken. Some parents reflected:

"“I feel like punishing him is a bit draconian, it is going to happen anyway…and I am trying to minimize the damage…”. (Mother of a 17 year-old boy)"

"“I don’t think I would punish her, I don’t think that would work, but I would let her know that I was really disappointed”. (Father of a 16 year-old girl)"

A number of parents recalled ‘one-off’ incidents where their child had come home drunk or been drunk when they were collected from a party. The most common reaction among parents in both groups was disappointment, with anger being secondary. Several parents also said their greater concern or reaction was in relation to their child’s dishonesty rather than the drinking itself. In cases where children had been drunk, parents commonly stated that punishment wasn’t necessary because the sense of shame and the hangover were enough to teach their child a lesson.

Some research suggests that parents who lack effective skills in family management have less capacity to protect their children from negative influence of peers [21]. Adolescents with poor family management are more likely to engage in heavy episodic drinking [22] and be exposed to risky situations such as socialising with peers who are drinking [23]. In contrast, Ryan et al. found no relationship between rules about alcohol and either early initiation or later drinking levels [6].

#### Model responsible drinking and attitudes towards alcohol

Parents consistently cited their family background as a strong influence on their drinking behaviours and attitudes. Several participants cited parents’ or grandparents’ alcoholism having ‘put them off’; that it influenced their choice to become non-drinkers or very light drinkers. A majority of parents reported that they and/or their partners drank relatively frequently, often having one or two drinks after work or when hosting friends or family for dinner. This was simply reported as the normal household pattern of drinking, with only a small minority of parents indicating that they consciously modeled a particular approach to drinking or level of consumption – *“we really try not to drink around the kids much”* (Mother of a 13 year-old girl and a 15 year-old boy).

Several parents cited heavy drinking by their children’s fathers or step-parents as influences on divorce or separation. According to the parents, these behaviours had mixed impacts on their children. Some children expressed anger and were deterred from drinking by their parents’ or step-parents’ heavy drinking, others have become heavy drinkers themselves. This contrast is highlighted in comments such as *“The oldest was never curious as a child – she was probably put off by seeing Dad come home drunk too often”* (Mother of 14, and 16 year-old boys and an 18 year-old girl) and *“the older boys don’t seem to have been put off at all by seeing their dad drunk so often – they both drink [on] most if not all weekends”* (Mother of 15, 18 and 20 year-old boys and separated from what the mother described as an alcoholic husband). The influence of older siblings was also important for both parents and adolescents, which is consistent with evidence from large cohort studies [24,25].

While there was some matching between parents’ behaviour and the guidelines (not getting drunk around children or modeling having a good time without alcohol), most parents discussed their own alcohol consumption as personal preference rather than a plan to model certain behaviour. In discordance with the guidelines, many parents cited associating having a drink with stress relief, or referred to the “need” for a glass of wine in conversation with their adolescent: *“I just tell them that Mum’s had a stressful day and needs a wine”* (Mother of 14 and 16 year-old boys and an 18 year-old girl), *“I joke that I need a glass of wine to cope with them [children]”* (Mother of a 14 year-old boy and a 16 year-old girl). Some parents also referred to asking older adolescents to pick them up when they had been drinking.

The varied influences of modeling and family of origin mirror the literature, with Ryan et al. citing mixed evidence, but reporting that overall, parental modeling of drinking is associated with early initiation to drinking and increased later use [6]. The influence of parental modeling of alcohol consumption though, is likely to be mediated by a range of other factors including parenting style and behaviour management, peer influence, and expectations associated with alcohol consumption [26-28]. An inverse relationship has been demonstrated between normative alcohol use by parents (frequency and volume) and measures of positive parenting [29].

#### Talk to your child about alcohol

Many parents were careful to deliver clear messages to their children about alcohol, some presenting the context of use (e.g. drinking large amounts when in public and potentially exposed to violence or other risks) as the danger rather than the alcohol itself; *“I do a lot of talking to about alcohol – it’s not cool to be drunk…the impact on the liver, the brain, etc etc”* (Mother of 14 and 18 year-old boys and a 16 year-old girl). Others *“wait until [their] children bring up the issue with [them]”* and have only discussed alcohol in the context of their adolescent raising the issue. Several parents indicated that an incident with drinking or comment about others’ drinking was the initial trigger for discussions about alcohol and the household rules.

Several studies of alcohol-specific communication between parents and adolescents have reported complex associations with adolescent drinking behaviours. In a prospective cohort study, Van der Vorst et al. found that frequent discussions about alcohol were counterproductive to reducing heavy drinking among male adolescents [30]. However, this result should be interpreted in light of the possibility that discussions about alcohol typically occur after the child has started to drink [14]. Parental discussions and attitudes may also be influenced by parents’ own consumption patterns. A Dutch study found that parents who experienced alcohol-related problems were more likely to engage in alcohol-specific communication with their adolescent children, but that this did not significantly affect the risk of adolescent alcohol use or alcohol-related problems [31]. Ryan et al. reported insufficient evidence of a relationship with alcohol-specific communication, but strong evidence that good general communication is associated with both delayed alcohol initiation and lower levels of later use [6].

### Guidelines followed less often

#### Delay your adolescent’s introduction to drinking alcohol

The guidelines recommend delaying the use of alcohol for as long as possible; not giving children any alcohol when they are under 15 years of age, and delaying their first drink for as long as possible. Overall, parents’ behaviour matched poorly with this suggestion. Most of the parents did not supply alcohol to their children for consumption outside the home, and reported making it clear to their children that they would not do so. Many, however, saw a certain inevitability that their children would be exposed, or gain access to alcohol prior to turning 18. One mother said *“I can’t prevent the drinking, I have to accept that it will happen, but I won’t supply it”.* Another who refused to supply her son with alcohol knew his older sister was giving it to him – *“the transaction happens in front of me”.* Parents’ sense of powerlessness to prevent alcohol consumption was common: *“we do not supply alcohol to them…I know that my 17 year-old is drinking at parties, and he comes home smelling of alcohol…I can’t stop him”* (Mother of a 17 year-old boy)*.*

A sense of inevitability was also expressed by those parents who did supply their children with alcohol. One parent, reflecting on having provided her sons with a six pack of beer to take to parties from age 16 said *“I don’t like doing it but I see it as a way of keeping a limit on it”.* Even parents who claimed to have reasonably strict rules and close monitoring of their children admitted they would allow some drinking from around age 16 or 17; *“I would let them drink with a small group of friends…in a safe environment. I wouldn’t want them to be social outcasts from their peer group”* (Mother of a 15 year-old boy and younger girl). Other parents refer to their impression of the law and have adopted *“you don’t drink until you’re 18”* as their rule. However, many of these parents were also aware that their children were drinking from a younger age and expressed a sense of powerlessness to prevent it. One mother, when discussing drinking at a party with her 17 year-old son, told him *“we won’t allow you, but we can’t stop you”.*

The provision of sips or tastes of alcohol at home was generally regarded as quite separate to the provision of alcohol for consumption outside the home. Comments such as *“we have given him half a glass of beer with us, but basically they are not allowed to drink”* (Mother of 15 year-old boy) and *“might be allowed to have a sip, but we never give them alcohol…I would never give them alcohol to take to a party”* (Mother of a 13 year-old girl and 16 year-old boy) capture the views of many parents. Even some of the strictest parents, who regarded themselves as *“disciplinary…not their friend”* (Mother of 16 and 17 year-old boys) and report having strict household rules, have allowed their older adolescents to try *“a little sip”* of alcohol. Two main opposing views were expressed; one that children should not drink at all before they turn 18, and the other that a gradual, safe introduction would likely prevent excessive, harmful drinking later on. The latter view was articulated clearly by the father of a 16 year-old boy who explained *“over the next two years we will be introducing him to alcohol. We don’t want him to be suddenly 18 and go on a rampage”* (Father of a 15 year-old boy)*.* Interestingly, one of the strictest proponents of the former view reported that her son *“has indicated that he is going to have an 18*^*th*^*and get drunk”* (Mother of a 17 year-old boy)*,* highlighting a potential desire for those who are deprived from trying alcohol to rebel when the opportunity arises.

Some parents said they provided their children with small amounts of alcohol from a young age to avoid demonising alcohol or fuelling curiosity that may lead to rebellion. This is consistent with reports of parents providing children with alcohol in order to control consumption and encourage safe use [11]. Views on the conditions under which parents would supply alcohol were consistent with research conducted in New Zealand where parents generally expressed responsible attitudes on the subject [4]. It should be noted that in that research, involving 872 teenagers aged 13-17 and 748 parents in the same school communities, there were large discrepancies between teenagers’ and parents’ reports concerning supply of alcohol for unsupervised drinking [32]. The authors suggested that the discrepancy may reflect differences in understanding of the survey questions but was more likely the result of socially desirable responses from parents.

Ryan et al. report associations of the provision of alcohol, with both early initiation and higher levels of drinking [6]. A US study examined permissibility of drinking at home during the senior year of school (aged 17-18) as a potential predictor of young women’s alcohol consumption early in their college careers. The study reported that those women who had been allowed to drink at home, whether during meals or with friends, reported more frequent heavy episodic drinking in their first semester of college, but those allowed to drink with friends drank more per drinking occasion [33]. Lundborg also reported that parents’ willingness to supply was not linked with more responsible drinking patterns in adolescents [34]. These results are in keeping with those of our recent study with high school students in Australia, in which parental supply under all conditions was associated with an increased probability of risky drinking [35]. Parental supply of alcohol for consumption when parents were present was no longer significantly associated with risky drinking once other confounders (including supply for consumption under other adult supervision, or no supervision, as well as peer-related variables) were controlled for. Those findings suggest that while some parents give their children alcohol to ensure a safe introduction, this type of supply may not protect young people from engaging in risky drinking. In another study with Australian adolescents, Dietze et al found that risky drinking was more likely among those supplied with alcohol from sources other than parents [36].

#### Prepare your adolescent to deal with the influence of peers

Parents expressed great difficulty in protecting their children from the influence of peers. Generally, parents viewed the choice of peer group as a significant influence upon their adolescents’ behaviours, but parents felt that they had minimal influence over these choices. One parent said of her 16-year-old son: *“he’s probably more influenced by the pack leader than by us [parents] now”.* Parents of adolescents who didn’t drink frequently cited close friendship groups that *“just aren’t interested”* or are *“not into that”.* Some parents commented on their children’s peer groups: *“the circle of friends that he hangs with are all good kids”* (Father of a 15 year-old boy), *“the group are the same, they do not drink”* (Father of a 16 year-old girl), “*he is going through with a peer group that have been drinking for a couple of years”* (Mother of a 17 year-old boy).

Those who chose not to drink or who had not yet begun experimenting with alcohol often commented on the behaviour of *“other kids at parties and at their school”* and expressed negative attitudes towards drunkenness among peers at age 14 to 16. One parent relayed her daughter’s description of drunken 14 year-olds as *“disgusting and stupid”.* A clear differentiation was made between close friendship groups and the wider group of ‘kids at school’. One parent discussed the dramatic positive change in the behavior of her two sons when they moved areas and therefore moved away from the peer groups that she perceived to be a bad influence on them.

Parents also frequently commented on personality differences between children and indicated that some children just weren’t interested; and even between siblings, differences often existed. Several parents recalled their children calling to be collected, or coming home early from parties where there was a lot of alcohol consumption because they felt uncomfortable. Discussions about perceptions of alcohol and drinking appeared to be more difficult for parents when they knew their child’s peers had engaged in alcohol consumption.

Recent work with Australian parents supports the sentiments that parents expressed in this study. Ward and Snow reported on parents’ intentions to supply their children with alcohol, suggesting that such behaviours may be a reflection of the normalization of alcohol use in Australia [37]. In another Australian study, parents cited peer influence as a major contributor to adolescent alcohol use, particularly among younger adolescents [38]. The strong influence of peer groups and normative beliefs [39] represents a great challenge for parents in influencing their adolescent’s perceptions toward alcohol and alcohol consumption.

#### Enlist the support of other parents

Several parents described “*making an effort”* to know other parents. This was commonly referred to as an effort rather than something that happened naturally or easily. One parent whose son had started a new school in a new area in year 10 (age 15) discussed the fact that she knew very few of his friends or the parents of his friends, and that she felt *“less comfortable with that”.* It was a common theme among parents that they didn’t know many of the other parents, and it was not easy to change this situation. Most parents knew some of their children’s friends, but this was expressed more as a product of their child’s personality and extra-curricular activities than a deliberate effort. For example: *“They have a community of kids…90% are elite athletes…most weekends we spend with these kids…do not drink at all because they are so focused on their goals”.*

When asked if they would call parents they didn’t know to discuss alcohol being supplied at a party, parents had very mixed views. Some indicated that they would feel more comfortable doing so if they did not know the parents, others suggested that doing so would not be helpful with parents who obviously had differing values to their own, and others still put the responsibility on their adolescent children to make their own informed decisions and deal with the situation as they saw fit. The difficulty of engaging with other parents was evident in the following comments:

"“It can be who they hang around. I think it depends on the other parents’ morals. Some people are more liberal than others. I think with high school they mix with a lot of people that are not in your social circle…” (Mother of 16 year-old girl);"

"“If you ring the police…or confront them, it puts you in an awkward position…I think the best thing you can do is talk to your children and…just ask them to be sensible” (Mother of a 17 year-old boy)."

The guidelines recommend that parents ‘build a support network with other parents’, but this was not common among the parents in the study. Parents did refer to friends with older or similar aged children and were often influenced in their parenting decisions by strategies they had observed among their own peers. Interestingly though, it was often the pressure imposed by other parents’ behaviour that was often raised:

"“…my friend gave her 13 year-old a sip…and my daughter looked at me and asked the question…I probably would have told her ‘no’ if she was on her own but I didn’t want her to feel left out” (Mother of a 13 year-old girl)."

"“…all the parents were saying that they gave their kids alcohol” (Mother of a 17 year-old boy)."

"“I don’t want my child to miss out on things. We are teaching our children to be individuals but we, as parents have issues with the peer pressure” (Mother of a 13 year-old boy and a 16 year-old girl)."

The difficulties expressed by parents in this study reinforce the need for support which has been reported by parents [38] and recognized as a need in other Australian studies [37,40]. It is expected that parents’ decisions about supplying their children with alcohol are likely to be influenced by their knowledge of the behaviours of other parents [41]. It seems however, that parents have limited contact with the parents of their children’s peers, and are therefore not likely to have an accurate understanding of their behaviours and attitudes. This represents a key opportunity to provide assistance to parents in reducing adolescent alcohol use.

### Other common themes

#### Developmental stage: the social clock and peer influence

Many of the comments parents made regarding their relationships with, and discipline of their children, referred to the developmental stage, or to different approaches or reactions depending upon the age of the child. A common pattern of change in children’s attitudes towards alcohol was described by most parents. When very young, their children were curious about alcohol and interested in tasting their parents’ drinks. In late primary school and early adolescence, many children adopt quite a negative attitude toward drinking and tend not to be interested in or to enjoy the taste of alcoholic drinks. At a point that appears to differ among children according to their personalities, peer groups and family experiences, they then develop an interest in drinking. This issue is highlighted by comments such as “*I’m always telling them that being drunk is not cool but that message was taken on board more at 16 than at 17, nearly 18”* (Mother of a 17 year-old boy)*.* Further, when faced with hypothetical scenarios involving children of different ages, parents indicated that they felt they had more control of their children’s behaviour at 16 than at close to 18; “*I couldn’t stop the 17 year-old but I’d be willing to say no at 16”* (Mother of a 17 year-old boy)*.*

These comments are in keeping with the concept of the *social clock*, which refers to the age at which it is considered acceptable in a particular society, to reach various milestones or transitional events [41]. The social clock is also reflected in comments about drinking at home. Many parents allowed their young children to have sips or tastes of alcohol from their own drinks but cited children’s lack of interest in late primary and early secondary school. Some parents clearly stated their children were not allowed to have a drink, whether it be a sip with a family meal or at a special occasion. Other parents had not faced this situation or even considered their ‘rules’ because their children had not expressed interest in drinking. This is in keeping with comments that adolescents *“bypass the taste and drink to get drunk”.* Many parents discussed the fact that their children didn’t enjoy the taste of alcohol, but would drink *“lolly water”* (alcopops) for the effect it gave. The influence of peers may exacerbate the social clock phenomenon, with peer groups seemingly progressing together past each milestone.

### Laws about adolescents and alcohol

When discussing household rules about alcohol or the approach adopted, parents rarely referred to any externally provided guidelines. While several parents spoke about the ‘law’, they generally referred to their understanding that drinking under 18 years of age is illegal. This was a commonly held misinterpretation of the law in Australia. One parent questioned the message her son received from a school talk which he interpreted as *“…I’m allowed to give him alcohol”.* The parents generally had little understanding of the law surrounding alcohol consumption in private settings and secondary supply. Several parents described their impressions of the law, indicating clear misinterpretation e.g., one mother who reported that she would give her 16 year-old son two light beers to take to a party, and that she gave her 10 year-old a glass of orange juice with a splash of champagne in it for a birthday treat said *“I know legally I’m not supposed to”*. Many parents referred to drinking “underage” and interpreted the law as *“when they are 18 they can drink”*. Similarly, parents had little knowledge of guidelines regarding adolescent alcohol consumption, saying their decisions were based on their own values and discussions with friends, rather than external guidance.

### Limitations

Convenience sampling was used to recruit parents for this study, potentially limiting the representativeness of the sample and generalizability of results. Further, while the guidelines were deliberately not explicitly mentioned in interviews, it may be that parents were more aware of them than was apparent, or that parents' attitudes and adherence to them could have been more accurately explored had we prompted discussion of them specifically. It is a strength of the study, however, that by avoiding explicit mention, the parents were less likely to be biased by any pressure to provide what they may have thought we wanted to hear. Finally, the study relies purely on the perceptions and reports of parents, without direct link to adolescent behaviour. Parents' perceptions of their control over their adolescents' behaviour or the impact of their household rules and approaches on their adolescents' drinking were not explicitly explored. The primary aim of this study was to investigate the views and behaviours of a heterogeneous group of parents. There would be value in further research to estimate the impact of adherence to the guidelines on adolescent alcohol consumption.

## Conclusion

This study has shown that while parenting styles and approaches to adolescent alcohol consumption vary, the intentions of parents are generally consistent. Most parents report using strategies they believe will minimise harm to their adolescents and promote their healthy and safe development in a society where experimentation with alcohol is seen as inevitable. Parents’ approaches, however, don’t always match the recommendations of the parenting guidelines for adolescent alcohol use. While the guidelines address key areas of concern for parents, parents’ awareness of them, and level of adherence to the suggested approaches is low. Consistent with the lack of knowledge of the NHMRC guidelines for reducing the harm of drinking for Australian adults [42], it seems that parents’ knowledge of these guidelines is poor.

There were two key areas in which the approaches used by parents deviated most from the guidelines. Firstly, the recommendation to delay the introduction to drinking alcohol was not consistently adopted by parents. Even parents who reported being very strict with, and closely monitoring their children, allowed them to try small amounts of alcohol, and in later adolescence, allowed them to drink in a safe environment with friends. Similarly, while parents discussed drinking as ‘uncool’ and ensured that their adolescents knew to call to be collected if they were in uncomfortable situations, parents found it difficult to protect their children from peer influence and were disinclined to stop their children from attending parties where alcohol was consumed. Several parents said they would allow some drinking to prevent their children from feeling socially isolated.

There was no obvious relationship between the rules set by, or ‘strictness’ of, parents and the drinking behaviours or attitudes to drinking among adolescents. To some extent, this validates the sense of inevitability expressed by many parents. It was common for parents to express powerlessness to prevent their adolescents from drinking and being exposed to alcohol, and also, to express their own sense of peer pressure, with the knowledge that their children’s friends were drinking and that other parents may allow some drinking and supply their children with alcohol.

Further dissemination of the guidelines to parents may be the first step in providing assistance, but it is likely that parents would require support to effectively adopt them. With many parents having referred to the guidance of friends, and with the guidelines recommending the development of a support network of parents, establishing and strengthening parental networks may help parents to implement positive behaviours.

## Competing interests

None

## Authors’ contributions

CG and KK prepared the interview protocol. CG and a research assistant conducted the interviews and thematic analysis of data, and CG prepared the manuscript. KK reviewed the interview notes and thematic analysis and assisted with manuscript preparation.

## Pre-publication history

The pre-publication history for this paper can be accessed here:

http://www.biomedcentral.com/1471-2458/12/491/prepub

## Supplementary Material

Additional file 1 Interview schedule used with parents. Click here for file
