# Supplementary material for: Parent attitudes, family dynamics and adolescent drinking: qualitative study of the Australian parenting guidelines for adolescent alcohol use
Source: BMC Public Health. 2012 Jun 29;12:491. doi: 10.1186/1471-2458-12-491 (PMC3461436; doi:10.1186/1471-2458-12-491)
Supplement: Additional file 1 — Interview schedule used with parents. [file 1471-2458-12-491-S1.doc]

**INTERVIEW PROTOCOLS**

**Sources of supply for teenage binge drinking**

**Questions will be modified based on the direction of the discussion.**

Introduction

Interview

Welcome and introduction.

Before we start I would like to clarify a few things about how the interview will proceed. There are no right or wrong answers to any of the questions I am going to ask, and you will not be judged in any way. You were invited today because you have knowledge that may help us design an intervention to prevent alcohol-related harm of young people.

The information provided by you will be treated confidentially and you will not be identified personally in any results or reports arising from our discussion. I also need to ask if you mind me recording today’s conversation so I can make some notes afterwards. The recording won’t be fully transcribed. Some quotes might be used in reports, but won’t be linked with you individually. I can send you a copy of my notes so you can review and edit them afterwards. Is that ok?

Today’s discussion is purely voluntary and you can withdraw at any time. The Information Statement you received contains contact details if you have any problems with this research. I also have some information here about support services available to you if you’d like to speak to someone about any concerns. In the unlikely event that I hear about something in the discussion that suggests that you or another person might be in danger or exposed to violence then we have an obligation to report that. Do you have any questions before we start?

Questionnaire:

Pass participant the demographic and drinking behaviour questionnaire – ask them to complete it.

Semi-structured interview:

What is normal for your family in relation to alcohol consumption?

- How often is alcohol consumed?
- Alcohol at celebrations etc.

Do or have your children expressed an interest in alcohol/sharing drinks with you?

- Curiosity
- Children’s attitudes when younger – now as teenagers.

How would you describe your relationship with you teenage children at the moment?

- Respect/trust
- Friendship
- Discipline
- Tension/disagreement

Have you discussed the rules about alcohol for your children with your partner/spouse/other parent?

- Do you agree about the rules?
- If not, how do you overcome this?

What are the rules about alcohol for your children?

- Have you explicitly discussed these with your child?
- Have they tried alcohol?
- When are they allowed to drink alcohol?

Do you ever give your children alcohol? Under what circumstances do you give your child alcohol?

- Special occasions
- At home
- To take to a party
- Never

If you give your children alcohol to take to a party, what are your reasons for doing this?

Do you think your children respect the rules you have set in place? How can you tell if they are breaking them? What are the consequences for this?

- Are your children following the rules?
- If not, how have they been punished?
- What was their reaction to the consequences you set?

Do you think your children could talk to you about their alcohol consumption, and what happens as a result?

Do you think there is pressure to supply alcohol to your children? Where do you think this comes from?

Scenarios:

Your 16 year old child is going to a party where the parents have supplied alcohol. What do you do?

Your child is going to their best friend’s 18^th^ birthday, but they are not yet 18. The parents supervising the party have not supplied alcohol, and your child is asking you to get them some alcohol to take with them to the party. What do you do?

On a Sunday after apparently staying over at a friend’s house to watch movies the night before, your fifteen year old is showing signs of a hangover (you think they have been throwing up, are obviously very tired, not eating etc). What do you do?

5 minute summary to end:

**Question:**

Did I correctly describe what was discussed today?

Close

Thank you for your comments today. You’ve provided me with some really useful information which we’ll use to help design a project to tackle some of the issues faced by young people regarding alcohol and its harms.

**Question:**

Is there anything we should have talked about today but didn’t?
